# Variability of human fasted venous plasma metabolomic profiles with tourniquet induced hemostasis

**DOI:** 10.1038/s41598-021-03665-2

**Published:** 2021-12-27

**Authors:** Sarita Devi, Roshni M. Pasanna, Nikhil Nadiger, Santu Ghosh, Anura V. Kurpad, Arpita Mukhopadhyay

**Affiliations:** 1 Division of Nutrition, St. John’s Research Institute, St. John’s National Academy of Health Sciences, Sarjapur Road, Bangalore, 560034 India; 2 Department of Biostatistics, St. John’s Medical College and Hospital, St. John’s Research Institute, St. John’s National Academy of Health Sciences, Bangalore, India

**Keywords:** Biochemistry, Biological techniques, Molecular biology, Medical research, Molecular medicine

## Abstract

Venous plasma metabolomics is a potent and highly sensitive tool for identifying and measuring metabolites of interest in human health and disease. Accurate and reproducible insights from such metabolomic studies require extreme care in removing preanalytical confounders; one of these is the duration of tourniquet application when drawing the venous blood sample. Using an untargeted plasma metabolomics approach, we evaluated the effect of varying durations of tourniquet application on the variability in plasma metabolite concentrations in five healthy female subjects. Tourniquet application introduced appreciable variation in the metabolite abundances: 73% of the identified metabolites had higher temporal variation compared to interindividual variation [Intra-Class Correlation (ICC) > 0.50]. As such, we recommend tourniquet application for minimal duration and to wait for 5 min with the needle in situ after removing the tourniquet, to reduce hemostasis-induced variability and false flags in interpretation.

## Introduction

Metabolomics, or the identification and measurement of metabolites in biological samples, has become an invaluable means for generating new knowledge in terms of markers for prognosis and diagnosis of human pathophysiology as well as of new leads in understanding causal pathways^1–4^. Of the various possible sources of samples from human subjects for metabolomic assessments, components of blood (plasma and serum), remain the most relevant and therefore, most studied type of sample^5^. However, unlike metabolomics performed with samples generated from in vitro or animal model experiments that are inherently less heterogenous, using plasma or serum metabolomics as a tool in human translational research is subject to the inherent interindividual variability between subjects due to genetic and environmental factors and their interactions. An additional source of variation is pre-analytic factors. Controlling for such variability introducing pre-analytic factors would be key to improving reproducibility of high impact human metabolomic findings such as the use of sarcosine as a biomarker for progression of prostate cancer^6,7^. Here, fasting status, type of collection tube, presence of haemolysis, time taken and temperature of sample during processing, temperature and duration of storage of processed samples, and freeze–thaw cycles have been assessed^8–12^. Unknown and unaccounted for variation arising due to pre-analytic factors could lead to overestimation of interindividual variation or even intraindividual variation, if the samples have been collected at multiple time points in a longitudinal fashion. However, Agueusop et al.^13^ reported remarkable stability of the human serum metabolome over a 4 weeks’ time period, when the samples were collected on 3 different days under stringently controlled conditions.

An additional, unstudied and potentially significant variation might arise from local tissue hemostasis during venous blood collection due to variable tourniquet application times and associated haemostasis. We could not identify any study that has investigated this effect on metabolomic readouts in the plasma. Therefore, we explored the effect of different durations of tourniquet-induced local hemostasis on fasted venous plasma metabolomic profiles of South Asian Indian women, for a deeper understanding of tourniquet-time related precautions required for reproducible untargeted human metabolomic studies. Our hypothesis was that the number of metabolites differing in abundance from the control, unrestricted blood flow condition would increase in step with the increased time of tourniquet-induced local hemostasis.

## Results

Socio-demographic characteristics, anthropometric measurements and metabolic profiles of the study participants are summarized in Table 1. All 05 subjects were apparently healthy, pre-menopausal females (age range 26.7–33.3 years). The BMI of the subjects ranged from normal to overweight (21.6–27.3 kg/m^2^). The subjects were normoglycemic (mean fasting glucose: 88.2 ± 5.2 mg/dL) and normotensive [mean systolic blood pressure and diastolic blood pressure (mmHg): 107.4 ± 5.8 and 84.2 ± 7.4 respectively].

**Table 1:** Subject characteristics and metabolic profile of the 05 study subjects.

	Mean ± SD
Age (years)	31.4 ± 2.7
BMI (kg/m^2^)	23.9 ± 2.4
Systolic blood pressure (mmHg)	107.4 ± 5.8
Diastolic blood pressure (mmHg)	84.2 ± 7.4
HbA1c (%)^a^	5.4 ± 0.2
Fasting plasma glucose (mg/dL)	88.2 ± 5.2
Fasting serum insulin (mU/L)	10.5 ± 5.5
Serum cholesterol (mg/dL)^a^	166.6 ± 19.6
Serum triglycerides (mg/dL)^a^	78.6 ± 38.8
Serum creatinine (mg/dL)^a^	0.7 ± 0.1

Data are mean ± SD.

^a^Fasted blood sample.

**Figure 1:**
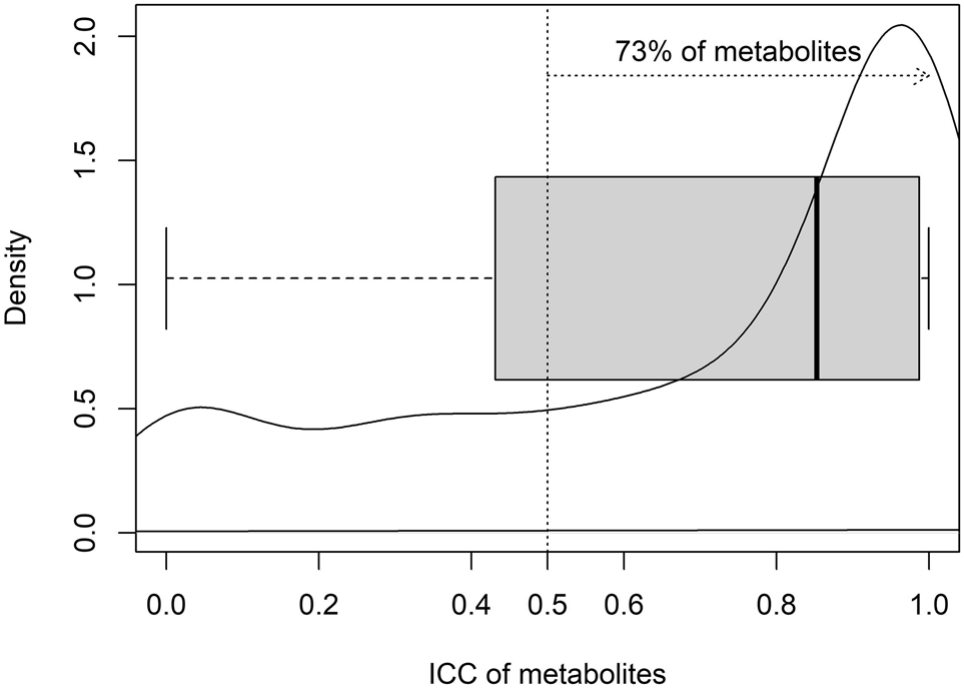
Distribution of temporal variability, measured by Intra-Class Correlation (ICC) of the 353 metabolites, identified as differentially abundant between the 4 collection time points [T1, T2, T4: 1, 2 and 4 min, respectively, after tying the tourniquet and NT (no tourniquet with sample collection 5 min after removal of the tourniquet)] in 05 study participants. The bold dark vertical line of the box-whisker plot superimposed on the density indicates the median ICC, the left and right sides of the rectangle are indicative of 25th and 75th percentiles and the whiskers measure the 95% confidence interval. The vertical dotted line at 0.50 ICC has been included to visualize the proportion of metabolites accounting for temporal variability > 50% of total variation of each metabolite.

**Figure 2:**
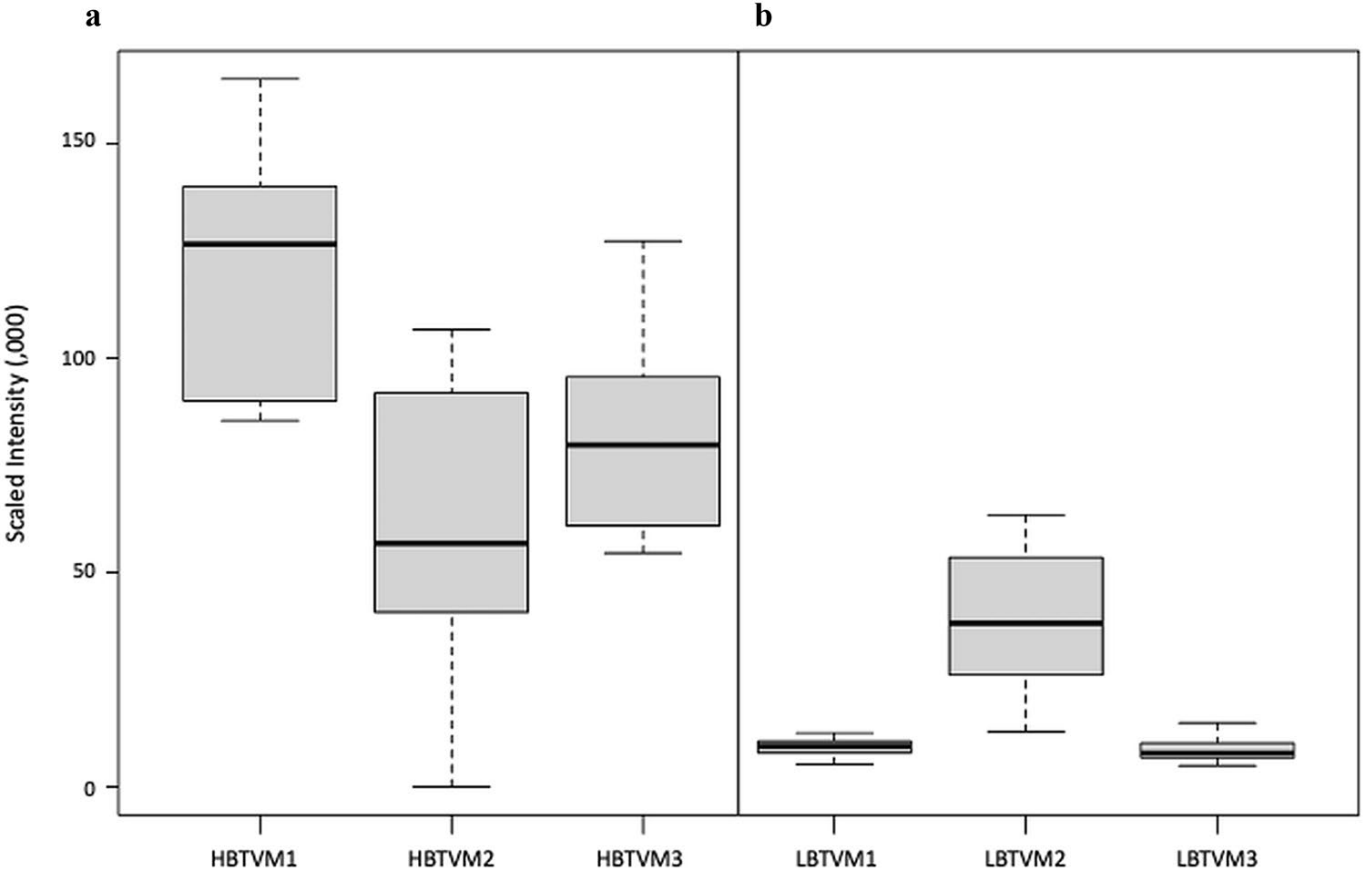
Box-whisker plots representing pareto-scaled intensities of selected High- and Low-Between Timepoint Variability Metabolites (HBTVM and LBTVM, respectively). (**a**) HBTVM1: 1-Linoleoyl-sn-glycero-3-phosphocholine, HBTVM2: 1-Oleoyl-sn-glycero-3-phosphocholine, HBTVM3: 1-Palmitoyl-sn-glycero-3-phosphocholine. (**b**) LBTVM1: pro-pro-pro, LBTVM2: l-Isoleucine, LBTVM3: l-Glutamic acid. The bold dark vertical line of the box-whisker plot indicates the median CV, the top and bottom ends of the rectangle are indicative of 25th and 75th percentiles and the whiskers measure the 95% confidence interval.

A total of 353 metabolites were identified in the study plasma samples by the Compound Discoverer software (version 3.1.0.305, ThermoFisher Scientific, Vanquish Flex Binary, Waltham, MA, USA). Since our primary aim was to identify and quantitate the variation, if any, in the relative abundance of plasma metabolite across the 4 collection time points in all the study participants, we decomposed total variability into within time point variation (inter-individual variation) and between time point variation (temporal variation) (Supplementary Table 1). The temporal variation in the relative abundance of the identified metabolites between the 4 plasma collection time points, i.e., 1, 2 and 4 min after tying the tourniquet (T1, T2, T4 respectively) and free flowing blood with no tourniquet (NT, which was the collection made 5 min after the removal of the tourniquet,) in 5 study participants is summarized in Fig. 1. In this, 73% of the identified metabolites had higher temporal variation compared to interindividual variation, as evident from Intra-Class Correlation (ICC) > 0.50, ICC being the proportion of temporal variation over total (temporal + inter-individual) variation. Interestingly, among all identified metabolites, 24 metabolites did not exhibit any temporal variation. The inter-individual variation for these 24 metabolites ranged between 11.4 to 98.1% (Supplementary Table 1). Box plots representing pareto-scaled intensities of three of these metabolites (pro-pro-pro, l-Isoleucine and l-Glutamic acid) and of three selected metabolites with high between timepoint temporal variability (1-Linoleoyl-sn-glycero-3-phosphocholine, 1-Oleoyl-sn-glycero-3-phosphocholine and 1-Palmitoyl-sn-glycero-3-phosphocholine) is presented in Fig. 2. The raw and Pareto-scaled intensities of the 353 metabolites for all study subjects and sample collection time points is available in Supplementary Tables 2 and 3 respectively.

**Figure 3:**
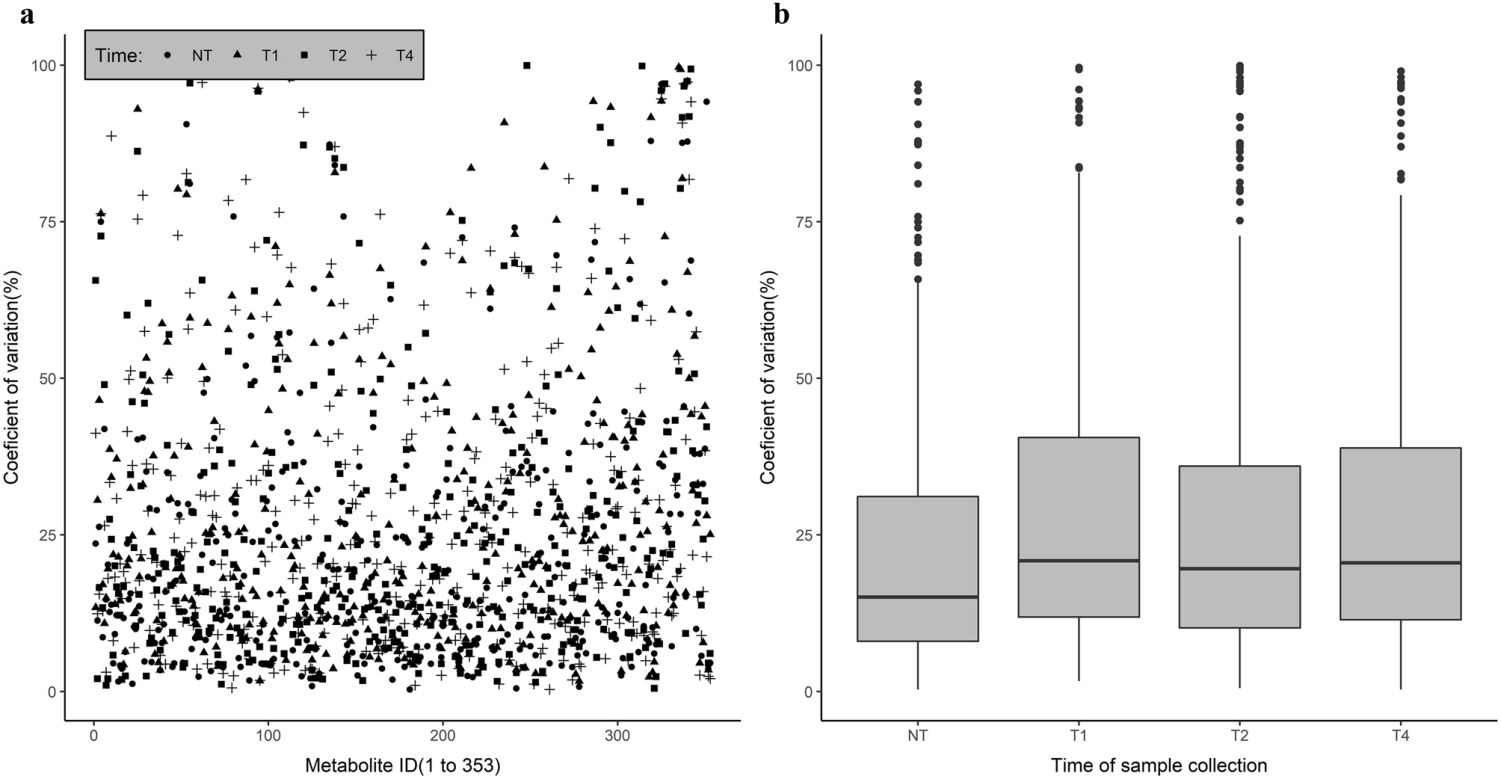
The coefficient variation (CV) (representing inter-individual variability) of each metabolite at four sample collection time points [T1, T2, T4: 1, 2 and 4 min, respectively, after tying the tourniquet and NT (no tourniquet with collection 5 min after removal of the tourniquet)] in 05 study participants. The CVs were calculated as the ratio of median absolute deviation about median to median of the unscaled abundance (peak intensities) of respective metabolites and plotted for all 353 metabolites, identified as differentially abundant between the 4 collection time points (**a**) and as box-whisker plot (**b**) for each of the 4 collection time points summarizing the same feature by more interpretable manner. The bold dark horizontal line of the box-whisker plot indicates the median CV, the top and bottom ends of the rectangle are indicative of 25th and 75th percentiles and the whiskers measure the 95% confidence interval.

In order to further understand the effect of tourniquet-induced haemostasis on variability in metabolite abundances, we derived the interindividual variability of each metabolite at the 4 collection time points by coefficient variation (CV) calculated as the ratio of median absolute deviation (MAD) about median to median of the raw abundance of respective metabolites (Fig. 3). The CVs ranged up to 100% and when evaluated at each collection time point, the median CV was least at NT (15.11%, Table 2).

**Table 2:** Distribution of interindividual variability of metabolite abundance of the 353 metabolites, measured as the Coefficient of Variations (CVs) by collection time points [T1, T2, T4: 1, 2 and 4 min, respectively, after tying the tourniquet and NT (no tourniquet with collection 5 min after removal of the tourniquet)] in 05 study participants. The CVs were calculated as the ratio of median absolute deviation about median to median of the unscaled abundance (peak intensities) of respective metabolites.

Sample collection time point	N	Mean	Median	SD	Quartile 1	Quartile 3
NT	353	22.58	15.09	20.17	8.01	31.12
T1	353	28.28	20.88	22.20	11.88	40.54
T2	353	27.49	19.61	24.36	10.17	36.00
T4	353	28.74	20.56	23.47	11.46	38.88

T1, T2, T4 are 1, 2 and 4 min respectively, of time elapsed after tying the tourniquet; NT is no tourniquet, where blood sample collection occurred 5 min after removal of the tourniquet.

## Discussion

Obtaining accurate and reproducible insights when using metabolomics tools in clinical research requires a critical evaluation of technical factors contributing to the overall variability of metabolomics profiles. We systematically interrogated the effect of duration of application of a tourniquet during collection of blood from healthy subjects, on the variability in metabolite concentrations using an untargeted metabolomics approach. We observed considerable temporal variability in relative abundance of the identified metabolites. The intra-class correlations, that measured the temporal variability in relation to the overall variability, were more than 0.50 for 73% of the identified metabolites (Fig. 1) and the mode was at ~ 0.90. Therefore, the duration of tourniquet application is an important factor that induces variability in measurement of abundance of the metabolites.

The inter-individual variation in metabolite abundances was higher amongst the metabolites with least temporal variation compared to those with highest temporal variation. This is plausible in light of the observation that amongst the 24 metabolites without any temporal variation were sesamex (a synergist for insecticides)^14^, valdecoxib (a non-steroidal anti-inflammatory drug)^15^ and vanillin (primary component of vanilla bean extract)^16^. These are exogenous metabolites, whose presence in the plasma is likely indicative of an individual’s consumption/exposure to the relevant exogenous sources.

With regard to time point of blood collection to minimize introducing variation due to tying of the tourniquet, the ideal situation would be to not use the tourniquet at all. However, considering the need for using the tourniquet to find the vein for blood collection in group of subjects where finding the vein is otherwise difficult, the duration of tourniquet application should be minimal and one should wait for 5 min with the needle in situ after removing the tourniquet, before blood sampling. As a rule, the shorter the duration of application of torniquet, the better the accuracy in the measurement of metabolites.

A strength of our study is that we minimized inter-individual variation in plasma metabolomic profiles by carefully including subjects as similar to each other as possible, in terms of age (range 26.7–33.3 years), sex (all pre-menopausal females), ethnicity (South Asian Indian) and geographical location (Bangalore, India), as well as by collecting all samples within a one-hour time period in the morning, after a pre-defined fasting time period (11–12 h) These factors have been earlier reported to contribute to variability in human blood (plasma/serum) metabolomic profiles (age^17–20^, sex^19,20^, geographical location^21–23^, fasting/mealtime^24^ and time of day when sample is collected^24^). The small number of participants, with blood sampling at relatively few discrete time points, are limitations.

To conclude, for untargeted human plasma metabolomics studies, we recommend that blood should be collected without the application of a tourniquet for needle insertion. If this is needed, for example, when finding a vein is difficult, blood should be collected within 1 min of applying the tourniquet; if not, blood should be collected from the in situ needle at least 5 min after removing the tourniquet. With common sense, we also recommend making other a priori decisions on the exact standard operating procedure for the subject’s fasting/fed state, duration of fasting, timing of the day for collection of sample, type of sample collection tubes, duration of tourniquet application during sample collection, time–temperature-duration of storing the samples before processing, steps of sample processing including sample aliquot sizes to minimize freeze–thaw cycles, time–temperature-duration of storing the samples after processing and before freezing, duration of sample storage under frozen condition, number of permissible freeze–thaw cycles and also of the steps for sample processing after thawing, for metabolomics analysis, at the start of a study. A uniform, practical and rigid standard operating procedure that addresses these variability-inducing factors is a prerequisite for improving reproducibility and therefore, the relevance of plasma or serum metabolomics as a tool for interrogating human health and disease.

## Methods

### Study design and inclusion criteria

Apparently healthy female subjects (18–60 year old) volunteering to participate in the study were recruited at the Division of Nutrition, St. John’s Research Institute, St. John’s Medical College and Hospital, Bangalore. Exclusion criteria were: age outside 18–60 years range, not willing to participate in the study, were participating in other studies, had tested positive for hepatitis (HBsAg) or HIV, needed chronic or daily medical therapy (connective tissue diseases, inflammatory bowel disease, active tuberculosis, symptomatic heart disease), had serious pre-existing clinical conditions.

#### Ethical approval and informed consent

Ethical approval for the study had been sought and obtained from the Institutional Ethics Committee of St. John’s Medical College and Hospital, Bangalore. Explanation of the study protocol was done in the language understood by the participants. Informed, signed consent from the participants was obtained at recruitment. All relevant guidelines and regulations were followed while carrying out the study protocol.

#### Socio-demographic data and medical history

Well-structured questionnaires were provided to the participants and explained by trained study personnel for obtaining detailed socio-demographic data. Information on recent medical history (3 months prior to recruitment) of the participants, and on medications and nutritional supplements being currently taken by them was obtained and duly recorded.

#### Anthropometric and vital signs measurements

A digital scale, to a precision of 0.1 kg, was used to weigh the subjects, in minimal clothing, while the height of the subjects were recorded to the nearest 0.1 cm. Measurement and recording of blood pressure and pulse was done appropriately by trained study personnel.

### Sample collection and clinical chemistry

An overnight fasting (11–12 h fasting) blood sample (10 mL) was collected in EDTA (BD Vacutainer^®^, Becton, Dickinson and Company, Franklin Lakes, NJ) tubes between ∼ 0830 and 0930 h by arm (antecubital vein) venepuncture. Four collection time points [T1, T2, T4: 1, 2 and 4 min respectively, after tying the tourniquet, and NT (no tourniquet): collection 5 min after removal of the tourniquet] were included for each study participant.

Samples were transferred immediately and stored in an ice box (< 1.5 h of collection). Post-centrifugation (1166 rcf, 10 min, 4 °C) in a cooling centrifuge (REMI C-23 BL Cooling centrifuge, Mumbai, India), the plasma was aliquoted in cryovials and stored at − 80 °C until analysis. Glucose, total cholesterol, triglycerides and creatinine (Beckman Coulter AU480 Chemistry Analyzer, Beckman Coulter, Brea, CA), insulin (ROCHE Hitachi Elecsys 2010 Chemistry Analyzer, Basel, Switzerland) and haemoglobin A1c (Siemens Dimension XPand Plus Analyzer, Siemens, Erlangen, Germany) levels were measured using standard plasma and serum clinical chemistry assays.

### High-resolution accurate-mass (HRAM) data analysis

Plasma samples were processed by following similar protocol as described earlier^25^. Briefly, plasma samples (100 μL) were spiked with an internal standard (IS) of a ^2^H-labelled amino acid mixture (20 μL, 1 ng/mL; U-^2^H labelled amino acid mix > 97% purity; Cambridge Isotope Laboratories, Massachusetts, USA) and deproteinised using chilled organic solvent (8:1:1, acetonitrile: methanol: acetone). Samples were vortex-mixed and incubated at 4 °C for 30 min before centrifugation at 20,000 rcf for 20 min in a refrigerated centrifuge (5810 R, Eppendorf, Eppendorf AG, Hamburg, Germany). Supernatants were dried at 40 °C in a vacuum concentrator (Labconco, USA) and dried extracts were reconstituted in acetonitrile/water (1:1). Untargeted metabolomics analysis was performed on a high-resolution accurate-mass (HRAM) platform consisting of an ultra-high pressure liquid chromatograph (UHPLC, Thermo Scientific, Vanquish Flex Binary, Waltham, MA, USA) coupled to an orbitrap based mass spectrometer (Q Exactive, Thermo Scientific, San Jose, USA). The mass spectrometer was calibrated by using a positive ion calibration solution (Pierce LTQ Velos ESI Positive Ion Calibration Solution, ThermoFisher Scientific, Waltham, MA, USA) on daily basis before starting an analytical sequence consisting of solvent blanks, pooled quality control (QC) samples, which included six technical replicates of a pool of aliquots derived from the study plasma samples^26^ and study plasma samples. Separation of the metabolites was achieved by using a Zorbax Eclipse plus-C18 column (150 × 2.1 × 1.8 micron, Agilent Technologies, Santa Clara, CA, USA) maintained at 40 °C. Other LC–MS/MS related method parameters are similar to that described earlier^25^.

Raw data files acquired through the Xcalibur software^27^ (version 4.1, ThermoFisher Scientific, MA, USA) were initially processed by using the Compound Discoverer software^28^ (version 3.1.0.305, ThermoFisher Scientific, Waltham, MA, USA) for positive polarity with an untargeted metabolomics workflow as described earlier^25^ to find and identify the differences between samples. The workflow used the adaptative curve model with 2 min maximum shift, 5 ppm mass tolerance, and 3 S/N (signal/noise) threshold for retention time alignment. Peak detection required less than 5 ppm mass error for extracted ion chromatograms with a minimum peak intensity of 1,000,000. [M + H]^+ 1^ was set as base ion with consideration for other adducts. Peaks were required to have a width at half height less than 0.5 min and a minimum of 5 scans. The maximum element count for isotope pattern modelling was C_90_H_190_Br_3_Cl_4_K_2_N_10_Na_2_O_15_P_3_S_5_. All detected compounds were grouped across samples with 5 ppm mass error and 0.2 min retention time shift. Missing peaks (not detected initially) in a given sample were determined using the Fill Gaps node algorithm with 5 ppm mass error and 1.5 S/N threshold with real peak detection. The Fill Gaps node calculates the area of missing chromatographic peaks as follows: matching detected ions based on expected *m/z* and retention time regardless of adduct assignment, re-detecting peaks at lower thresholds, simulating peaks based on expected *m/z*, and imputing spectrum noise based on detection limit values. Further, QC based area correction is applied for instrument drift using the cubic spline regression model. Each compound was required to be detected in at least 50% of QC runs with a Relative Standard Deviation (RSD) less than 30%. Compound identification was achieved by using mzCloud (ddMS2) and ChemSpider (formula or exact mass) and similarity searches for all compounds with ddMS2 data was done by using mzCloud and mzLogic algorithm applied to rank order ChemSpider results. The pre-processed data were exported to .xlsx files for further statistical analysis.

### Statistical analyses

Anthropometric and metabolic profile data were presented as mean ± SD and median with interquartile range (IQR). Metabolite abundance data was scaled using the pareto scaling method^29^ and variance of metabolites were decomposed into inter and intra individual variabilities by standard linear random effects model. The proportion of intra-individual variability over total was assessed by Intra-Class Correlation (ICC) for each metabolite. A chi-square test was performed to test whether temporal variability is different from zero within estimation algorithm of random effects model. We considered acceptable false positive rate at most 5%. Further, using the unscaled raw abundance (peak intensities) of the metabolites, the inter individual variability of each metabolite at four collection time points were derived by coefficient variation (CV) calculated as the ratio of median absolute deviation (MAD) about median to median of the raw abundance of respective metabolites. The statistical software R version 4.0.2 was used for the analysis^30^ (R Core Team, 2020, Vienna, Austria).

## Supplementary Information

Supplementary Table 1.

Supplementary Table 2.

Supplementary Table 3.

## Data Availability

The datasets generated and analysed during the current study that support the findings of this study are available from the corresponding author on reasonable request.
