# Supplementary material for: Variability of human fasted venous plasma metabolomic profiles with tourniquet induced hemostasis
Source: Sci Rep. 2021 Dec 27;11:24458. doi: 10.1038/s41598-021-03665-2 (PMC8712516; doi:10.1038/s41598-021-03665-2)
Supplement: Supplementary file 1 — Supplementary Table 1. [file 41598_2021_3665_MOESM1_ESM.docx]

**Supplementary Information**

**Manuscript title: Variability of human fasted venous plasma metabolomic profiles with tourniquet induced hemostasis**

Sarita Devi^1^, Roshni M. Pasanna^1^, Nikhil Nadiger^1^, Santu Ghosh^2^, Anura V. Kurpad^1^ and Arpita Mukhopadhyay^1,*^

^1^Division of Nutrition, St. John’s Research Institute, St. John’s National Academy of Health Sciences, Bangalore, India;

^2^Department of Biostatistics, St. John’s Medical College and Hospital, St. John’s Research Institute, St. John’s National Academy of Health Sciences, Bangalore, India.

**Corresponding author:*

Division of Nutrition

Division of Nutrition, St. John’s Research Institute, St. John’s National Academy of Health Sciences, Sarjapur Road, Bangalore, India. PIN: 560034

Phone: +91-80-49467000

Fax: +91-80-25501088

Email address: [arpitam@sjri.res.in](mailto:arpitam@sjri.res.in)

**Supplementary Table 1**. Within time point variation (inter-individual variation) and between time point variation (temporal variation) for the 354 metabolites, identified as differentially abundant between the 4 collection time points [T1, T2, T4: 1, 2 and 4 minutes, respectively, after tying the tourniquet and NT (no tourniquet with collection 5 minutes after removal of the tourniquet)] in 05 study participants. Intra-Class Correlation (ICC) was measured as ratio of temporal variation to total variation (inter-individual and temporal variation). P values included are from chi-square test for variance of metabolite abundance.

| **Metabolite ID** | **Metabolite** | **Within Time point SD (Interindividual variation)** | **Between Time Point SD (Temporal variation)** | **P value** | **ICC** | **ICC(%)** |
| --- | --- | --- | --- | --- | --- | --- |
| 1 | a-Linolenic acid | 0.287 | 1.288 | 0.000 | 0.953 | 95.263 |
| 2 | Vigabatrin | 0.177 | 0.260 | 0.001 | 0.683 | 68.269 |
| 3 | Vanillin | 0.675 | 0.000 | 1.000 | 0.000 | 0.000 |
| 4 | Valylvaline | 0.161 | 1.160 | 0.000 | 0.981 | 98.117 |
| 5 | Valine | 0.156 | 0.118 | 0.099 | 0.365 | 36.480 |
| 6 | Valdecoxib | 0.601 | 0.000 | 1.000 | 0.000 | 0.000 |
| 7 | Uric acid | 0.136 | 0.094 | 0.141 | 0.324 | 32.431 |
| 8 | Trimethadione | 0.168 | 0.111 | 0.171 | 0.301 | 30.145 |
| 9 | Trigonelline | 0.145 | 0.692 | 0.000 | 0.958 | 95.818 |
| 10 | Triethylamine | 1.391 | 0.528 | 0.551 | 0.126 | 12.589 |
| 11 | Triethanolamine | 0.163 | 0.095 | 0.248 | 0.252 | 25.234 |
| 12 | Tributyl phosphate | 0.068 | 1.927 | 0.000 | 0.999 | 99.877 |
| 13 | Triadimefon | 0.281 | 0.228 | 0.073 | 0.397 | 39.689 |
| 14 | Trans-3-Indoleacrylic.acid | 0.186 | 0.122 | 0.172 | 0.301 | 30.060 |
| 15 | Trans-2-Dodecenoylcarnitine | 0.128 | 0.507 | 0.000 | 0.940 | 94.007 |
| 16 | Timoprazole | 0.087 | 1.944 | 0.000 | 0.998 | 99.800 |
| 17 | Tetrahydrothiophene-2-carboxylic acid | 0.154 | 0.110 | 0.124 | 0.339 | 33.929 |
| 18 | Threo-Sphingosine,(-)- | 0.715 | 0.988 | 0.002 | 0.656 | 65.613 |
| 19 | Theobromine | 0.192 | 0.933 | 0.000 | 0.959 | 95.937 |
| 20 | Tetraacetylethylenediamine | 0.189 | 0.529 | 0.000 | 0.887 | 88.725 |
| 21 | Tert-Butyl.3-amino-1-methyl-2,3-dioxopropylcarbamate | 0.283 | 0.342 | 0.006 | 0.593 | 59.262 |
| 22 | Taurine | 0.388 | 0.217 | 0.274 | 0.238 | 23.834 |
| 23 | Sumatriptan | 0.104 | 2.144 | 0.000 | 0.998 | 99.763 |
| 24 | Succinimide,N-ethyl- | 0.194 | 0.168 | 0.054 | 0.427 | 42.668 |
| 25 | Stigmatellin Y | 0.700 | 1.275 | 0.000 | 0.768 | 76.849 |
| 26 | Stearoylcarnitine | 0.207 | 1.028 | 0.000 | 0.961 | 96.100 |
| 27 | Stearamide | 0.512 | 1.724 | 0.000 | 0.919 | 91.896 |
| 28 | Sesamex | 0.780 | 0.000 | 1.000 | 0.000 | 0.000 |
| 29 | Salinosporamide B | 0.348 | 0.363 | 0.017 | 0.521 | 52.122 |
| 30 | S-Allyl-L-cysteine | 0.441 | 0.563 | 0.004 | 0.620 | 62.049 |
| 31 | Ricinoleic Acid | 0.283 | 1.701 | 0.000 | 0.973 | 97.310 |
| 32 | Rhodamine.6G | 0.739 | 0.251 | 0.622 | 0.103 | 10.321 |
| 33 | Pro-pro-pro | 0.291 | 0.000 | 1.000 | 0.000 | 0.000 |
| 34 | Propionylcarnitine | 0.148 | 0.387 | 0.000 | 0.873 | 87.313 |
| 35 | Prolylleucine | 0.146 | 0.148 | 0.020 | 0.509 | 50.928 |
| 36 | Pregabalin | 0.165 | 0.332 | 0.000 | 0.802 | 80.189 |
| 37 | Poly THF n8 | 0.115 | 1.633 | 0.000 | 0.995 | 99.505 |
| 38 | Poly THF n7 | 0.059 | 1.812 | 0.000 | 0.999 | 99.894 |
| 39 | Platelet-activating factor | 0.171 | 3.420 | 0.000 | 0.998 | 99.752 |
| 40 | Pizotifen | 0.168 | 2.117 | 0.000 | 0.994 | 99.371 |
| 41 | Pivagabine | 0.198 | 0.033 | 0.896 | 0.027 | 2.653 |
| 42 | Piperine | 0.159 | 0.663 | 0.000 | 0.945 | 94.538 |
| 43 | Pipecolic acid | 0.632 | 0.000 | 1.000 | 0.000 | 0.000 |
| 44 | Physovenine | 0.212 | 0.871 | 0.000 | 0.944 | 94.388 |
| 45 | Pheophorbide A | 0.119 | 1.238 | 0.000 | 0.991 | 99.077 |
| 46 | Phenylglyoxylic acid | 0.913 | 0.000 | 1.000 | 0.000 | 0.000 |
| 47 | Phenmetrazine | 0.238 | 0.168 | 0.133 | 0.331 | 33.120 |
| 48 | Phaseic acid | 0.783 | 0.799 | 0.020 | 0.510 | 51.035 |
| 49 | Pentoxyverine | 0.411 | 0.291 | 0.130 | 0.334 | 33.411 |
| 50 | Pentabamate | 0.217 | 0.310 | 0.001 | 0.671 | 67.099 |
| 51 | PEG n11 | 0.196 | 0.000 | 1.000 | 0.000 | 0.000 |
| 52 | PEG n10 | 0.215 | 0.000 | 1.000 | 0.000 | 0.000 |
| 53 | PC(20:5(5Z,8Z,11Z,14Z,17Z)/P-18:1(11Z)) | 0.634 | 1.586 | 0.000 | 0.862 | 86.237 |
| 54 | PC(18:3(9Z,12Z,15Z)/18:2(9Z,12Z)) | 0.857 | 1.175 | 0.002 | 0.653 | 65.269 |
| 55 | PC | 0.811 | 1.545 | 0.000 | 0.784 | 78.425 |
| 56 | Palmitoylcarnitine | 0.191 | 2.086 | 0.000 | 0.992 | 99.171 |
| 57 | Palmitoyl ethanolamide | 0.111 | 1.889 | 0.000 | 0.997 | 99.659 |
| 58 | Palmitelaidic acid methyl ester | 0.062 | 1.780 | 0.000 | 0.999 | 99.879 |
| 59 | Oxohongdenafil | 0.185 | 0.000 | 1.000 | 0.000 | 0.000 |
| 60 | O-ureido-D-serine | 0.138 | 0.083 | 0.223 | 0.267 | 26.694 |
| 61 | O-nonanoylcarnitine | 0.163 | 0.354 | 0.000 | 0.825 | 82.509 |
| 62 | Oleamide | 1.002 | 1.900 | 0.000 | 0.782 | 78.240 |
| 63 | Octodrine | 0.729 | 0.000 | 1.000 | 0.000 | 0.000 |
| 64 | N-Undecanoylglycine | 0.410 | 0.000 | 1.000 | 0.000 | 0.000 |
| 65 | N-tetradecanoylsphinganine | 1.516 | 1.030 | 0.152 | 0.316 | 31.552 |
| 66 | N-Phenylacetylglutamine | 0.470 | 0.000 | 1.000 | 0.000 | 0.000 |
| 67 | NP-022512 | 0.806 | 0.149 | 0.870 | 0.033 | 3.312 |
| 68 | NP-020155 | 0.975 | 1.012 | 0.018 | 0.519 | 51.898 |
| 69 | NP-018660 | 0.584 | 1.256 | 0.000 | 0.822 | 82.231 |
| 70 | NP-013736 | 0.144 | 0.463 | 0.000 | 0.912 | 91.206 |
| 71 | NP-011548 | 0.068 | 1.127 | 0.000 | 0.996 | 99.641 |
| 72 | Noroxycodone | 0.142 | 1.127 | 0.000 | 0.984 | 98.444 |
| 73 | N-myristoylsphingosine-1-phosphocholine | 0.627 | 0.486 | 0.090 | 0.375 | 37.494 |
| 74 | N-Methylpyrrolidone | 0.138 | 0.015 | 0.950 | 0.012 | 1.245 |
| 75 | N-methylethanolamine phosphate | 0.171 | 0.248 | 0.001 | 0.679 | 67.858 |
| 76 | Nitrosoheptamethyleneimine | 0.179 | 0.391 | 0.000 | 0.826 | 82.589 |
| 77 | Nialamide | 0.820 | 0.000 | 1.000 | 0.000 | 0.000 |
| 78 | N-Hexanamide | 0.175 | 0.173 | 0.024 | 0.495 | 49.533 |
| 79 | N-Hexadecanoylsphinganine | 1.258 | 0.876 | 0.138 | 0.327 | 32.674 |
| 80 | N-Dodecanoylsphinganine | 1.405 | 0.924 | 0.170 | 0.302 | 30.194 |
| 81 | N-Butyl-1H-pyrazolo[3,4-d]pyrimidin-4-amine | 0.485 | 1.207 | 0.000 | 0.861 | 86.107 |
| 82 | Nadolol | 0.191 | 0.444 | 0.000 | 0.844 | 84.415 |
| 83 | N-ACETYLLYSINE | 0.193 | 0.195 | 0.021 | 0.505 | 50.534 |
| 84 | N-Acetyl-ala-ala-ala-methylester | 0.119 | 1.047 | 0.000 | 0.987 | 98.729 |
| 85 | N6-METHYLLYSINE | 0.177 | 0.054 | 0.685 | 0.084 | 8.429 |
| 86 | N~2~-Acetyl-L-ornithine | 0.201 | 0.261 | 0.003 | 0.627 | 62.723 |
| 87 | N,N-Bis(2-hydroxyethyl)dodecanamide | 0.571 | 1.496 | 0.000 | 0.873 | 87.282 |
| 88 | N-(tert-Butoxycarbonyl)-L-leucine | 0.137 | 0.139 | 0.021 | 0.507 | 50.710 |
| 89 | N(3)-(4-Methoxyfumaroyl)-2,3-diaminopropionic acid | 0.170 | 0.016 | 0.966 | 0.008 | 0.842 |
| 90 | Myxalamid A | 0.182 | 1.112 | 0.000 | 0.974 | 97.383 |
| 91 | Muscimol | 0.187 | 0.255 | 0.002 | 0.650 | 64.977 |
| 92 | Mucronine B | 0.244 | 0.922 | 0.000 | 0.935 | 93.468 |
| 93 | Moupinamide | 0.126 | 2.010 | 0.000 | 0.996 | 99.610 |
| 94 | Morphinone | 0.168 | 2.083 | 0.000 | 0.994 | 99.351 |
| 95 | Montelukast | 0.048 | 0.097 | 0.000 | 0.808 | 80.759 |
| 96 | Militarinone A | 0.161 | 3.393 | 0.000 | 0.998 | 99.775 |
| 97 | Oleoylcarnitine | 0.231 | 2.481 | 0.000 | 0.991 | 99.138 |
| 98 | N-(9Z-octadecenoyl)-glycine | 0.179 | 2.200 | 0.000 | 0.993 | 99.340 |
| 99 | Sphinganine 1-phosphate | 0.260 | 1.415 | 0.000 | 0.967 | 96.733 |
| 100 | METIPRANOLOL | 0.174 | 0.501 | 0.000 | 0.892 | 89.200 |
| 101 | Methylripariochromene A | 0.160 | 0.173 | 0.014 | 0.540 | 53.975 |
| 102 | Methylene blue | 1.121 | 1.196 | 0.015 | 0.532 | 53.216 |
| 103 | Methyl vinyl ketone | 0.146 | 1.848 | 0.000 | 0.994 | 99.377 |
| 104 | METHYL PALMOXIRATE | 0.271 | 1.839 | 0.000 | 0.979 | 97.873 |
| 105 | Methyl.indole-3-acetate | 0.172 | 0.847 | 0.000 | 0.960 | 96.044 |
| 106 | Methyl.2-(1,3,5-trihydroxy-4a-methyl-8-oxo-decahydronaphthalen-2-yl)prop-2-enoate | 0.822 | 0.000 | 1.000 | 0.000 | 0.000 |
| 107 | Meglutol | 0.182 | 0.142 | 0.089 | 0.376 | 37.621 |
| 108 | Medetomidine | 0.457 | 0.670 | 0.001 | 0.682 | 68.189 |
| 109 | Glutarimide | 0.131 | 0.124 | 0.033 | 0.471 | 47.078 |
| 110 | LysoPC(P-18:0) | 0.164 | 2.586 | 0.000 | 0.996 | 99.601 |
| 111 | LysoPC(22:5(7Z,10Z,13Z,16Z,19Z)) | 0.165 | 2.307 | 0.000 | 0.995 | 99.494 |
| 112 | LysoPC(20:5(5Z,8Z,11Z,14Z,17Z)) | 0.182 | 2.313 | 0.000 | 0.994 | 99.387 |
| 113 | LysoPC(18:3(9Z,12Z,15Z)) | 0.214 | 2.097 | 0.000 | 0.990 | 98.967 |
| 114 | L-Tyrosine | 0.122 | 0.196 | 0.000 | 0.721 | 72.072 |
| 115 | L-Theanine | 0.091 | 0.366 | 0.000 | 0.942 | 94.185 |
| 116 | L-Pyroglutamic acid | 0.181 | 0.116 | 0.188 | 0.289 | 28.898 |
| 117 | L-Phenylalanine | 0.152 | 0.097 | 0.190 | 0.288 | 28.792 |
| 118 | L-Isoleucine | 0.467 | 0.000 | 1.000 | 0.000 | 0.000 |
| 119 | Linoleyl carnitine | 0.133 | 2.664 | 0.000 | 0.998 | 99.753 |
| 120 | Linoleamide | 0.936 | 1.341 | 0.001 | 0.673 | 67.259 |
| 121 | L-Glutamic acid | 0.296 | 0.000 | 1.000 | 0.000 | 0.000 |
| 122 | Levalbuterol | 0.250 | 1.934 | 0.000 | 0.984 | 98.357 |
| 123 | Leu-Leu | 0.543 | 0.234 | 0.464 | 0.156 | 15.614 |
| 124 | Leucyl-leucyl-norleucine | 0.683 | 0.000 | 1.000 | 0.000 | 0.000 |
| 125 | Leonurine | 0.052 | 2.519 | 0.000 | 1.000 | 99.957 |
| 126 | Lentiginosine | 0.126 | 0.655 | 0.000 | 0.965 | 96.451 |
| 127 | Lawsone | 0.115 | 0.000 | 1.000 | 0.000 | 0.000 |
| 128 | L-alpha-Lysophosphatidylcholine | 0.146 | 3.329 | 0.000 | 0.998 | 99.809 |
| 129 | L-alpha-Glycerylphosphorylcholine | 0.047 | 2.650 | 0.000 | 1.000 | 99.968 |
| 130 | L-(+)-Citrulline | 0.128 | 0.253 | 0.000 | 0.795 | 79.529 |
| 131 | L-(+)-Alanine | 0.140 | 0.251 | 0.000 | 0.761 | 76.143 |
| 132 | L-(-)-Serine | 0.163 | 0.213 | 0.003 | 0.632 | 63.161 |
| 133 | L-(-)-Methionine | 0.156 | 0.104 | 0.162 | 0.308 | 30.796 |
| 134 | L(-)-Carnitine | 0.488 | 0.425 | 0.051 | 0.431 | 43.131 |
| 135 | Isothebaine | 0.244 | 2.547 | 0.000 | 0.991 | 99.089 |
| 136 | Isoquinoline | 0.177 | 0.863 | 0.000 | 0.960 | 95.964 |
| 137 | Isometheptene | 0.210 | 0.972 | 0.000 | 0.955 | 95.526 |
| 138 | Isocaffeine | 0.153 | 2.087 | 0.000 | 0.995 | 99.468 |
| 139 | Indole-3-acetic acid | 0.164 | 0.286 | 0.000 | 0.753 | 75.274 |
| 140 | Indane | 0.393 | 1.216 | 0.000 | 0.905 | 90.545 |
| 141 | Hypaphorine | 0.169 | 0.412 | 0.000 | 0.856 | 85.646 |
| 142 | Histamine | 0.199 | 1.495 | 0.000 | 0.983 | 98.251 |
| 143 | Hippeastrine | 0.159 | 1.035 | 0.000 | 0.977 | 97.698 |
| 144 | Hexanoylcarnitine | 0.133 | 0.295 | 0.000 | 0.832 | 83.164 |
| 145 | Hexadecasphing-4-enine-1-phosphate | 0.179 | 0.063 | 0.597 | 0.111 | 11.115 |
| 146 | Hexadecanamide | 0.414 | 2.169 | 0.000 | 0.965 | 96.483 |
| 147 | Hecogenin | 0.161 | 1.468 | 0.000 | 0.988 | 98.811 |
| 148 | Guvacine | 0.248 | 0.038 | 0.911 | 0.023 | 2.254 |
| 149 | Gly-l-pro | 0.188 | 0.252 | 0.003 | 0.643 | 64.342 |
| 150 | Glycyl-4-hydroxyproline | 0.189 | 0.203 | 0.014 | 0.537 | 53.664 |
| 151 | Glycocholic acid | 0.148 | 1.020 | 0.000 | 0.979 | 97.929 |
| 152 | Glycochenodeoxycholic acid | 0.137 | 0.812 | 0.000 | 0.972 | 97.224 |
| 153 | Glycerophospho-N-palmitoyl ethanolamine | 0.155 | 2.720 | 0.000 | 0.997 | 99.674 |
| 154 | Glyceraldehyde 3-phosphate | 0.115 | 0.037 | 0.650 | 0.095 | 9.470 |
| 155 | Glu-thr | 0.216 | 0.058 | 0.749 | 0.066 | 6.603 |
| 156 | Glu-ser | 0.633 | 0.000 | 1.000 | 0.000 | 0.000 |
| 157 | Gabapentin | 0.276 | 0.401 | 0.001 | 0.679 | 67.887 |
| 158 | Fluvoxamine | 0.162 | 3.679 | 0.000 | 0.998 | 99.807 |
| 159 | Ethylenediaminetetraacetic acid (EDTA) | 0.401 | 0.140 | 0.603 | 0.109 | 10.931 |
| 160 | Ethyl docosahexaenoate | 0.235 | 0.943 | 0.000 | 0.941 | 94.145 |
| 161 | Ethosuximide | 0.219 | 0.169 | 0.092 | 0.373 | 37.313 |
| 162 | N-Butyronitrile | 0.121 | 0.379 | 0.000 | 0.907 | 90.731 |
| 163 | Erucamide | 0.177 | 1.521 | 0.000 | 0.987 | 98.671 |
| 164 | Entadamide A | 0.525 | 0.756 | 0.001 | 0.675 | 67.498 |
| 165 | Eicosapentanoic acid | 0.162 | 1.351 | 0.000 | 0.986 | 98.576 |
| 166 | Ectoine | 0.180 | 0.155 | 0.053 | 0.427 | 42.721 |
| 167 | D-Pantothenic acid | 0.208 | 0.229 | 0.012 | 0.547 | 54.662 |
| 168 | DL-Tryptophan | 0.128 | 0.142 | 0.011 | 0.554 | 55.388 |
| 169 | DL-Thyroxine | 0.170 | 0.217 | 0.004 | 0.621 | 62.082 |
| 170 | DL-Stachydrine | 0.163 | 0.825 | 0.000 | 0.962 | 96.245 |
| 171 | DL-Lysine | 0.123 | 0.076 | 0.210 | 0.275 | 27.475 |
| 172 | DL-Homoserine | 0.277 | 0.304 | 0.012 | 0.546 | 54.637 |
| 173 | DL-Histidine | 0.133 | 0.319 | 0.000 | 0.851 | 85.130 |
| 174 | DL-Glutamine | 0.124 | 0.172 | 0.002 | 0.658 | 65.842 |
| 175 | DL-Carnitine | 0.153 | 0.065 | 0.477 | 0.152 | 15.154 |
| 176 | DL-Arginine | 0.150 | 0.277 | 0.000 | 0.774 | 77.409 |
| 177 | Dipropylene glycol dimethyl ether | 0.797 | 0.143 | 0.877 | 0.031 | 3.127 |
| 178 | Diisobutylphthalate | 0.077 | 3.055 | 0.000 | 0.999 | 99.936 |
| 179 | Dihydrothymine | 0.107 | 0.378 | 0.000 | 0.926 | 92.594 |
| 180 | Diethyl phthalate | 0.547 | 0.134 | 0.783 | 0.057 | 5.651 |
| 181 | Dicyclohexyl phthalate | 1.087 | 0.149 | 0.926 | 0.019 | 1.856 |
| 182 | D-Erythro-sphingosine.1-phosphate | 0.225 | 2.231 | 0.000 | 0.990 | 98.989 |
| 183 | Decanoylcarnitine | 0.134 | 0.342 | 0.000 | 0.867 | 86.719 |
| 184 | Deamino-alpha-keto-demethylphosphinothricin | 0.069 | 2.382 | 0.000 | 0.999 | 99.916 |
| 185 | D-Aspartatic acid | 0.210 | 0.082 | 0.531 | 0.133 | 13.274 |
| 186 | Diphenyl sulfoxide | 0.107 | 0.068 | 0.187 | 0.290 | 28.981 |
| 187 | D-(+)-Pyroglutamic.Acid | 0.325 | 0.000 | 1.000 | 0.000 | 0.000 |
| 188 | D-(+)-Proline | 0.171 | 0.388 | 0.000 | 0.838 | 83.788 |
| 189 | Cyprodenate | 0.958 | 1.486 | 0.001 | 0.706 | 70.616 |
| 190 | Cyclohexanone | 0.981 | 0.000 | 1.000 | 0.000 | 0.000 |
| 191 | Cyclo(phenylalanyl-prolyl) | 0.223 | 1.765 | 0.000 | 0.984 | 98.425 |
| 192 | Cyclo(deltaAla-L-Val) | 0.135 | 0.325 | 0.000 | 0.853 | 85.293 |
| 193 | Cxa-10 | 0.153 | 0.127 | 0.065 | 0.409 | 40.895 |
| 194 | Crotamiton | 0.077 | 1.929 | 0.000 | 0.998 | 99.841 |
| 195 | Creatinine | 0.295 | 0.201 | 0.150 | 0.317 | 31.725 |
| 196 | Creatine | 0.151 | 0.424 | 0.000 | 0.888 | 88.752 |
| 197 | Cis-5-Tetradecenoylcarnitine | 0.159 | 0.375 | 0.000 | 0.848 | 84.805 |
| 198 | Choline | 0.165 | 0.193 | 0.008 | 0.576 | 57.611 |
| 199 | Cetirizine | 0.033 | 3.368 | 0.000 | 1.000 | 99.990 |
| 200 | Capuride | 0.246 | 0.074 | 0.690 | 0.083 | 8.284 |
| 201 | Calcitriol | 0.181 | 1.615 | 0.000 | 0.988 | 98.754 |
| 202 | Buflomedil | 0.169 | 0.595 | 0.000 | 0.925 | 92.500 |
| 203 | Bis(methylbenzylidene)sorbitol | 0.289 | 0.491 | 0.000 | 0.743 | 74.310 |
| 204 | Bis(4-ethylbenzylidene)sorbitol | 0.729 | 2.244 | 0.000 | 0.904 | 90.441 |
| 205 | Bilirubin | 0.137 | 2.722 | 0.000 | 0.997 | 99.748 |
| 206 | Biacetyl | 0.243 | 0.148 | 0.217 | 0.270 | 27.032 |
| 207 | Betaine | 0.098 | 0.194 | 0.000 | 0.797 | 79.725 |
| 208 | Benzyl cyanide | 0.206 | 0.245 | 0.007 | 0.585 | 58.469 |
| 209 | Benserazide | 0.091 | 2.098 | 0.000 | 0.998 | 99.813 |
| 210 | Asarone | 0.162 | 0.157 | 0.029 | 0.482 | 48.177 |
| 211 | Arecaidine | 0.180 | 1.027 | 0.000 | 0.970 | 97.007 |
| 212 | Apronalide | 0.160 | 0.445 | 0.000 | 0.885 | 88.482 |
| 213 | Aniflorine | 0.159 | 0.330 | 0.000 | 0.812 | 81.159 |
| 214 | Anhalamine | 0.401 | 0.331 | 0.068 | 0.404 | 40.427 |
| 215 | Amino-levulinic acid | 0.162 | 0.075 | 0.413 | 0.176 | 17.574 |
| 216 | Alverine | 0.393 | 1.233 | 0.000 | 0.908 | 90.785 |
| 217 | Alpha-methylstyrene | 0.164 | 2.015 | 0.000 | 0.993 | 99.343 |
| 218 | Allopurinol | 0.291 | 0.369 | 0.004 | 0.616 | 61.596 |
| 219 | Aflatoxin B2 | 0.355 | 0.177 | 0.358 | 0.199 | 19.864 |
| 220 | Afegostat | 0.173 | 0.264 | 0.001 | 0.699 | 69.892 |
| 221 | Acetyl-L-carnitine | 0.147 | 0.123 | 0.062 | 0.413 | 41.324 |
| 222 | Acetylcholine | 0.154 | 0.199 | 0.004 | 0.624 | 62.443 |
| 223 | Aceglutamide | 0.429 | 0.326 | 0.099 | 0.365 | 36.508 |
| 224 | 9-Decenoylcarnitine | 0.131 | 0.387 | 0.000 | 0.896 | 89.638 |
| 225 | 9,12-Hexadecadienoylcarnitine | 0.191 | 0.370 | 0.000 | 0.790 | 79.035 |
| 226 | 9,10-Dihydroxystearic acid | 0.272 | 1.167 | 0.000 | 0.948 | 94.831 |
| 227 | 6-Methoxy-17-methyl-6,7,8,14-tetradehydro-4,5-epoxymorphinan-3-ol | 0.182 | 1.135 | 0.000 | 0.975 | 97.508 |
| 228 | 6,8-Dihydroxy-5,5-dimethyl-4,4a,5,6,7,8,10a,10b-octahydro-2H-benzo[e]furo[2,3,4-cd][2]benzofuran-9-one | 0.168 | 1.911 | 0.000 | 0.992 | 99.235 |
| 229 | 5-Oxonorleucine | 0.215 | 0.148 | 0.145 | 0.321 | 32.148 |
| 230 | 5-Nitro-o-toluidine | 0.154 | 1.266 | 0.000 | 0.985 | 98.534 |
| 231 | 5-Hydroxy-8-(hydroxymethyl)-6-isobutyryl-8-methyl-4-phenyl-2H,8H-pyrano[2,3-f]chromen-2-one | 0.162 | 0.140 | 0.055 | 0.425 | 42.463 |
| 232 | 5-fluoro AB-PINACA N-(4-hydroxypentyl) metabolite | 0.071 | 2.617 | 0.000 | 0.999 | 99.927 |
| 233 | 10-(boc-amino)decanoic acid | 0.180 | 0.418 | 0.000 | 0.843 | 84.279 |
| 234 | 1-Palmitoyl-sn-glycero-3-phosphocholine | 0.146 | 3.363 | 0.000 | 0.998 | 99.811 |
| 235 | 1,2-Dilinoleoyl-sn-glycero-3-phosphocholine | 0.700 | 2.391 | 0.000 | 0.921 | 92.115 |
| 236 | 5-[(9E,12E)-9,12,15-Hexadecatrienoyl]-5-hydroxy-4-methoxy-2(5H)-furanone | 0.147 | 0.461 | 0.000 | 0.907 | 90.704 |
| 237 | 5-[(5-Hydroxytetrahydro-2-furanyl)methyl]-1,3-benzenediol | 0.166 | 1.323 | 0.000 | 0.984 | 98.443 |
| 238 | 5,5'-Dihydroxy-4,4',8',8'-tetramethyl-4,5-dihydro-2'H,3H-spiro[furan-2,6'-[7]oxabicyclo[3.2.1]oct[3]en]-2'-one | 0.161 | 0.172 | 0.015 | 0.531 | 53.123 |
| 239 | 5-(6-hydroxy-6-methyloctyl)-2,5-dihydrofuran-2-one | 0.609 | 1.590 | 0.000 | 0.872 | 87.204 |
| 240 | 4-Sulfanyldihydro-3(2H)-furanone | 0.144 | 0.591 | 0.000 | 0.944 | 94.426 |
| 241 | 4-Ethoxy-m-anisaldehyde | 0.138 | 1.685 | 0.000 | 0.993 | 99.333 |
| 242 | 4-Ethoxy.ethylbenzoate | 0.058 | 2.166 | 0.000 | 0.999 | 99.929 |
| 243 | 4-amino-2-hydroxyamino-6-nitrotoluene | 0.121 | 1.797 | 0.000 | 0.996 | 99.551 |
| 244 | 4-Amino-1-[(2xi)-5-O-{hydroxy[(hydroxy{(2R)-2-[(18-methylicosanoyl)oxy]-3-[(9Z,11Z)-9,11-octadecadienoyloxy]propoxy}phosphoryl)oxy]phosphoryl}-beta-D-threo-pentofuranosyl]-2(1H)-pyrimidinone | 0.134 | 1.368 | 0.000 | 0.990 | 99.043 |
| 245 | Octylmethylamine | 0.978 | 0.479 | 0.369 | 0.194 | 19.356 |
| 246 | Myristoyl-L-carnitine | 0.148 | 0.266 | 0.000 | 0.764 | 76.432 |
| 247 | 2-(boc-amino)tetradecanoic acid | 0.129 | 0.303 | 0.000 | 0.846 | 84.591 |
| 248 | 1-Oleoyl-sn-glycero-3-phosphocholine | 0.150 | 3.526 | 0.000 | 0.998 | 99.821 |
| 249 | 4-{(Z)-[(4E,7Z,16Z,19Z)-1-Hydroxy-4,7,10,13,16,19-docosahexaen-1-ylidene]amino}butanoic.acid | 0.131 | 0.740 | 0.000 | 0.970 | 96.981 |
| 250 | 4-[(3-Acetamidopropyl)amino]butanoic acid | 0.205 | 0.210 | 0.020 | 0.513 | 51.279 |
| 251 | 4,6-Diamino-3-({3-amino-6-[1-(methylamino)ethyl]tetrahydro-2H-pyran-2-yl}oxy)-2-hydroxycyclohexyl.3-deoxy-4-C-methyl-3-(methylamino)pentopyranoside | 0.401 | 0.920 | 0.000 | 0.841 | 84.053 |
| 252 | 3-Thiomorpholinecarboxylic acid | 0.163 | 0.245 | 0.001 | 0.694 | 69.404 |
| 253 | 3-Oxalomalic acid | 0.092 | 0.085 | 0.038 | 0.458 | 45.821 |
| 254 | 3-hydroxyoctanoylcarnitine | 0.182 | 0.427 | 0.000 | 0.847 | 84.693 |
| 255 | 3-Hydroxy-cis-5-tetradecenoylcarnitine | 0.155 | 0.504 | 0.000 | 0.914 | 91.364 |
| 256 | 3-Buten-1-amine | 0.214 | 0.055 | 0.767 | 0.061 | 6.091 |
| 257 | 3-Aminopyrrolidine | 0.250 | 1.300 | 0.000 | 0.964 | 96.441 |
| 258 | p-Acetyltoluene | 0.690 | 0.785 | 0.010 | 0.564 | 56.363 |
| 259 | 3-[(3-Hydroxydecanoyl)oxy]-4-(trimethylammonio)butanoate | 0.159 | 0.488 | 0.000 | 0.905 | 90.453 |
| 260 | 3,5-di-tert-Butyl-4-hydroxybenzaldehyde | 0.071 | 2.046 | 0.000 | 0.999 | 99.880 |
| 261 | 3',5'-Cyclic IMP | 0.291 | 0.130 | 0.437 | 0.167 | 16.653 |
| 262 | 3,4-dehydrothiomorpholine-3-carboxylic acid | 0.503 | 0.373 | 0.108 | 0.355 | 35.468 |
| 263 | 3,.5-Tetradecadiencarnitine | 0.143 | 0.425 | 0.000 | 0.899 | 89.867 |
| 264 | 3-(3,4-Dimethyl-5-pentyl-2-furyl)propanoic acid | 0.145 | 1.154 | 0.000 | 0.984 | 98.444 |
| 265 | 3-(3,4,5-trimethoxyphenyl)propanoic acid | 0.160 | 1.677 | 0.000 | 0.991 | 99.099 |
| 266 | 3-(2,6-Dioxocyclohexyl)propanenitrile | 0.258 | 0.985 | 0.000 | 0.936 | 93.559 |
| 267 | 2-palmitoyl-sn-glycero-3-phosphocholine | 0.150 | 3.304 | 0.000 | 0.998 | 99.795 |
| 268 | 2-methylbutyrylcarnitine | 0.370 | 0.195 | 0.315 | 0.218 | 21.798 |
| 269 | 2-Methoxy-4-(3-oxobutyl)phenyl.N,N-dimethylcarbamate | 0.176 | 0.157 | 0.045 | 0.442 | 44.241 |
| 270 | 2-linoleoyl-sn-glycero-3-phosphoethanolamine | 0.160 | 2.672 | 0.000 | 0.996 | 99.642 |
| 271 | 2-Imino-1-isobutyl-5-oxo-1,5-dihydro-2H-dipyrido[1,2-a:2,3-d]pyrimidine-3-carbonitrile | 0.159 | 1.720 | 0.000 | 0.992 | 99.150 |
| 272 | 2-Deoxyhexopyranose | 0.819 | 0.000 | 1.000 | 0.000 | 0.000 |
| 273 | 2-Chromanone | 0.152 | 0.095 | 0.199 | 0.282 | 28.197 |
| 274 | 2-Ammonio-4-oxobutanoate | 0.167 | 0.185 | 0.012 | 0.550 | 55.017 |
| 275 | 2-Aminobutyric acid | 0.130 | 0.178 | 0.002 | 0.653 | 65.301 |
| 276 | 2'-Aminoacetophenone | 0.149 | 0.136 | 0.040 | 0.453 | 45.284 |
| 277 | Hexahydropyridine | 0.153 | 0.000 | 1.000 | 0.000 | 0.000 |
| 278 | 4,4'-Dioctyldiphenylamine | 0.065 | 2.315 | 0.000 | 0.999 | 99.922 |
| 279 | Anhydrovitamin A | 0.160 | 2.719 | 0.000 | 0.997 | 99.655 |
| 280 | 2-[Amino(carboxy)methyl]cyclopropanecarboxylic acid | 0.289 | 0.268 | 0.036 | 0.463 | 46.269 |
| 281 | 2-[2-(2,3-dihydro-1,4-benzodioxin-6-yl)-1,3-thiazol-4-yl]-1,3-benzothiazole | 0.371 | 0.241 | 0.178 | 0.296 | 29.608 |
| 282 | 2,4-Dimethylbenzaldehyde | 0.162 | 1.835 | 0.000 | 0.992 | 99.223 |
| 283 | 2,3-dinor-8-epi-prostaglandin.F1alpha | 0.155 | 1.786 | 0.000 | 0.993 | 99.250 |
| 284 | 2,3-Dimethoxy-5-methyl-6-(3-methyl-2-buten-1-yl)-1,4-benzenediol | 0.153 | 0.750 | 0.000 | 0.960 | 96.019 |
| 285 | 2,2,6,8-Tetramethyl-7,11-dioxatricyclo[6.2.1.0~1,6~]undec-4-ene | 0.840 | 1.343 | 0.000 | 0.719 | 71.896 |
| 286 | 2-(3-Ethyl-5-(4-methoxyphenyl)-1H-pyrazol-4-yl)phenol | 0.240 | 1.352 | 0.000 | 0.970 | 96.954 |
| 287 | 2-(2-Hydroxy-2-propanyl)-4-methoxy-7-methyl-2,3,6,7-tetrahydro-9H-furo[3,2-h]isochromen-9-one | 0.270 | 1.944 | 0.000 | 0.981 | 98.101 |
| 288 | 1-stearoyl-sn-glycero-3-phosphoethanolamine | 0.163 | 2.812 | 0.000 | 0.997 | 99.667 |
| 289 | 1-stearoyl-2-oleoyl-sn-glycero-3-phosphoserine | 0.419 | 1.276 | 0.000 | 0.903 | 90.285 |
| 290 | 1-oleoyl-sn-glycero-3-phosphoethanolamine | 0.151 | 2.380 | 0.000 | 0.996 | 99.600 |
| 291 | 1-O-Hexadecyl-lyso-sn-glycero-3-phosphocholine | 0.205 | 2.535 | 0.000 | 0.993 | 99.349 |
| 292 | 1-Methyl-4-[(8E)-10-methyl-6-methylene-8-undecen-2-yl]benzene | 0.146 | 0.643 | 0.000 | 0.951 | 95.134 |
| 293 | 1-linoleoyl-sn-glycero-3-phosphoethanolamine | 0.147 | 3.045 | 0.000 | 0.998 | 99.767 |
| 294 | 1-Linoleoyl-sn-glycero-3-phosphocholine | 0.137 | 3.540 | 0.000 | 0.999 | 99.851 |
| 295 | 1-hexadecyl-2-[(4Z,7Z,10Z,13Z,16Z,19Z)-docosahexaenoyl]-sn-glycero-3-phosphocholine | 0.307 | 0.994 | 0.000 | 0.913 | 91.322 |
| 296 | 1-hexadecanoyl-2-(4Z,7Z,10Z,13Z,16Z,19Z-docosahexaenoyl)-sn-glycero-3-phosphocholine | 0.612 | 1.432 | 0.000 | 0.846 | 84.551 |
| 297 | 1-heptadecanoyl-sn-glycero-3-phosphoethanolamine | 0.146 | 2.225 | 0.000 | 0.996 | 99.572 |
| 298 | 1-heptadecanoyl-sn-glycero-3-phosphocholine | 0.152 | 2.703 | 0.000 | 0.997 | 99.686 |
| 299 | 1-arachidonoyl-sn-glycero-3-phosphoethanolamine | 0.437 | 2.052 | 0.000 | 0.957 | 95.668 |
| 300 | 1-arachidonoyl-sn-glycero-3-phosphocholine | 0.155 | 3.184 | 0.000 | 0.998 | 99.763 |
| 301 | 1-Aminocyclohexanecarboxylic.acid | 0.167 | 0.319 | 0.000 | 0.785 | 78.468 |
| 302 | N-Boc-4-Piperidineethanol | 0.363 | 0.151 | 0.489 | 0.147 | 14.724 |
| 303 | N-myristoylglycine | 0.151 | 1.565 | 0.000 | 0.991 | 99.072 |
| 304 | Behenamide | 0.757 | 1.116 | 0.001 | 0.685 | 68.509 |
| 305 | 13S-hydroxyoctadecadienoic.acid | 0.177 | 1.529 | 0.000 | 0.987 | 98.676 |
| 306 | 12-oxo-ETE | 0.166 | 1.590 | 0.000 | 0.989 | 98.919 |
| 307 | 11-Nitro-1-undecene | 0.713 | 0.821 | 0.009 | 0.570 | 57.002 |
| 308 | 10-Chloro-1-heptadecene-4,6-diyne-3,9-diol | 0.115 | 1.279 | 0.000 | 0.992 | 99.197 |
| 309 | 1-{[(3S,4S,5R)-2,3,4-Trihydroxy-5-(hydroxymethyl)tetrahydro-2-furanyl]methyl}-2-pyrrolidinecarboxylic.acid.(non-preferred.name) | 0.568 | 0.298 | 0.320 | 0.216 | 21.559 |
| 310 | 1-[(9Z)-hexadecenoyl]-sn-glycero-3-phosphocholine | 0.147 | 3.181 | 0.000 | 0.998 | 99.788 |
| 311 | 1-[(8Z,11Z,14Z)-icosatrienoyl]-sn-glycero-3-phosphocholine | 0.213 | 3.304 | 0.000 | 0.996 | 99.588 |
| 312 | 1-[(1Z,9Z)-octadecadienyl]-sn-glycero-3-phosphocholine | 0.437 | 0.616 | 0.002 | 0.665 | 66.471 |
| 313 | 1-[(11Z)-octadecenoyl]-sn-glycero-3-phosphocholine | 0.144 | 3.153 | 0.000 | 0.998 | 99.791 |
| 314 | 1-[(11Z,14Z)]-icosadienoyl-sn-glycero-3-phosphocholine | 0.164 | 2.399 | 0.000 | 0.995 | 99.536 |
| 315 | 1,6-Dihydroxy-3-methoxy-10-methyl-9(10H)-acridinone | 0.084 | 2.161 | 0.000 | 0.998 | 99.848 |
| 316 | 1,5,6,7-tetrahydropteridin | 0.286 | 1.078 | 0.000 | 0.934 | 93.415 |
| 317 | 1-(4Z,7Z,10Z,13Z,16Z,19Z-docosahexaenoyl)-sn-glycero-3-phosphocholine | 0.220 | 2.286 | 0.000 | 0.991 | 99.079 |
| 318 | 1-(1Z-hexadecenyl)-sn-glycero-3-phosphocholine | 0.163 | 2.762 | 0.000 | 0.997 | 99.654 |
| 319 | {[[(4-Cyanobenzyl)amino](imino)methyl]amino}methanimidamide | 0.376 | 1.187 | 0.000 | 0.909 | 90.865 |
| 320 | Methyl 3-{[2-(4-nitrobenzoyl)hydrazino]sulfonyl}thiophene-2-carboxylate;.<U+0394>Mass:.39.3432.Da] | 0.116 | 1.906 | 0.000 | 0.996 | 99.631 |
| 321 | (+/-)12-HpETE;.<U+0394>Mass:.-51.0120.Da] | 0.056 | 2.131 | 0.000 | 0.999 | 99.930 |
| 322 | Alpha-Aminoadipic acid | 0.190 | 0.000 | 1.000 | 0.000 | 0.000 |
| 323 | (E)-p-Coumaric.acid | 0.122 | 0.191 | 0.001 | 0.708 | 70.844 |
| 324 | (E)-3,4,5-Trimethoxycinnamic.acid | 0.180 | 1.940 | 0.000 | 0.991 | 99.146 |
| 325 | (7Z,9Z,12Z,15Z,18Z,21Z)-7,9,12,15,18,21-Tetracosahexaenoic.acid | 0.240 | 1.891 | 0.000 | 0.984 | 98.419 |
| 326 | (5alpha,6beta)-3-Hydroxy-17-methyl-4,5-epoxymorphinan-6-yl.beta-L-glucopyranosiduronic.acid | 0.158 | 0.646 | 0.000 | 0.944 | 94.350 |
| 327 | (4S)-4-cyclohexyl-1-({[2-methyl-1-(propanoyloxy)propoxy](4-phenylbutyl)phosphoryl}acetyl)-L-proline | 0.170 | 2.025 | 0.000 | 0.993 | 99.297 |
| 328 | (4S)-4-[(2E)-2-Octenoyloxy]-4-(trimethylammonio)butanoate | 0.184 | 0.505 | 0.000 | 0.883 | 88.323 |
| 329 | (4E)-5-Hydroxy-4-{(2E,4E,6R)-1-hydroxy-6-[(3R,4R,6R)-6-hydroxy-1,4,8-trimethyl-2,9-dioxabicyclo[3.3.1]non-7-en-3-yl]-4-methyl-2,4-heptadien-1-ylidene}-2,4-dihydro-3H-pyrrol-3-one | 0.299 | 0.490 | 0.000 | 0.729 | 72.856 |
| 330 | (4E)-2-Amino-3-oxo-4-nonenoic acid | 0.159 | 0.307 | 0.000 | 0.789 | 78.867 |
| 331 | (2S)-4-Methyl-2-({[(3S,4S,5R)-2,3,4-trihydroxy-5-(hydroxymethyl)tetrahydro-2-furanyl]methyl}amino)pentanoic.acid.(non-preferred.name) | 0.510 | 0.284 | 0.277 | 0.237 | 23.650 |
| 332 | (2R)-3-Hydroxy-2-[(9Z,12E)-9,12-octadecadienoyloxy]propyl.2-(trimethylammonio)ethyl.phosphate | 0.161 | 3.548 | 0.000 | 0.998 | 99.796 |
| 333 | (2R)-3-{[(2-Aminoethoxy)(hydroxy)phosphoryl]oxy}-2-hydroxypropyl.pentadecanoate | 0.169 | 0.685 | 0.000 | 0.943 | 94.286 |
| 334 | (2R)-3-{[(2-Aminoethoxy)(hydroxy)phosphoryl]oxy}-2-hydroxypropyl.(9Z)-9-hexadecenoate | 0.161 | 2.334 | 0.000 | 0.995 | 99.529 |
| 335 | (2R)-2-[(4Z,7Z,10Z,13Z,16Z,19Z)-4,7,10,13,16,19-Docosahexaenoyloxy]-3-[(9Z)-9-hexadecenoyloxy]propyl.2-(trimethylammonio)ethyl.phosphate | 1.401 | 2.500 | 0.000 | 0.761 | 76.106 |
| 336 | (2R)-2-[(4Z,7Z,10Z,13Z,16Z,19Z)-4,7,10,13,16,19-Docosahexaenoyloxy]-3-[(6Z,9Z,12Z)-6,9,12-octadecatrienoyloxy]propyl.2-(trimethylammonio)ethyl.phosphate | 1.154 | 2.120 | 0.000 | 0.772 | 77.156 |
| 337 | (2R)-2-[(4Z,7Z,10Z,13Z,16Z,19Z)-4,7,10,13,16,19-Docosahexaenoyloxy]-3-[(5Z,8Z,11Z)-5,8,11-icosatrienoyloxy]propyl.2-(trimethylammonio)ethyl.phosphate | 0.601 | 1.475 | 0.000 | 0.858 | 85.768 |
| 338 | (2R)-1-{[(2-Aminoethoxy)(hydroxy)phosphoryl]oxy}-3-hydroxy-2-propanyl.(9Z,12Z,15Z)-9,12,15-octadecatrienoate | 0.163 | 1.928 | 0.000 | 0.993 | 99.292 |
| 339 | (2R)-1-{[(2-Aminoethoxy)(hydroxy)phosphoryl]oxy}-3-hydroxy-2-propanyl.(7Z,10Z,13Z,16Z,19Z)-7,10,13,16,19-docosapentaenoate | 0.157 | 2.509 | 0.000 | 0.996 | 99.608 |
| 340 | (2R)-1-{[(2-Aminoethoxy)(hydroxy)phosphoryl]oxy}-3-hydroxy-2-propanyl.(5Z,8Z,11Z,14Z,17Z)-5,8,11,14,17-icosapentaenoate | 0.142 | 2.089 | 0.000 | 0.995 | 99.538 |
| 341 | (2R)-1-{[(2-Aminoethoxy)(hydroxy)phosphoryl]oxy}-3-hydroxy-2-propanyl.(4Z,7Z,10Z,13Z,16Z)-4,7,10,13,16-docosapentaenoate | 0.159 | 1.327 | 0.000 | 0.986 | 98.590 |
| 342 | (2R)-1-{[(2-Aminoethoxy)(hydroxy)phosphoryl]oxy}-3-hydroxy-2-propanyl.(11Z)-11-icosenoate | 0.162 | 2.505 | 0.000 | 0.996 | 99.585 |
| 343 | (2E)-Hexadecenoylcarnitine | 0.233 | 2.035 | 0.000 | 0.987 | 98.702 |
| 344 | (2E,4Z)-N-Isobutyl-2,4-octadecadienamide | 0.341 | 2.205 | 0.000 | 0.977 | 97.664 |
| 345 | (2E,4E,12Z)-N-Isobutyl-2,4,12-octadecatrienamide | 0.454 | 1.390 | 0.000 | 0.903 | 90.342 |
| 346 | (25R)-3-Oxocholest-4-en-26-al | 0.177 | 1.508 | 0.000 | 0.986 | 98.646 |
| 347 | (2,7-Dimethyloctahydro-1H-cyclopenta[c]pyridin-4-yl)methanol | 0.509 | 0.219 | 0.464 | 0.156 | 15.641 |
| 348 | (1Z,9Z,12Z)-N-{(2R,6S,9S,11R,12R,13E,14aS,15S,16S,20S,21Z,23S,24Z,25aS)-2,8,11,12,14,15,22,25-Octahydroxy-6,20-bis[(1R)-1-hydroxyethyl]-23-[(1R)-1-hydroxy-2-(4-hydroxyphenyl)ethyl]-16-methyl-5,19-diox.o-2,3,5,6,9,10,11,12,14a,15,16,17,19,20,23,25a-hexadecahydro-1H-dipyrrolo[2,1-c:2',1'-l][1,4,7,10,13,16]hexaazacyclohenicosin-9-yl}-9,12-octadecadienimidic acid | 0.154 | 1.849 | 0.000 | 0.993 | 99.313 |
| 349 | (1R,9R)-5-(2-Methylphenyl)-11-(propylsulfonyl)-7,11-diazatricyclo[7.3.1.02,7]trideca-2,4-dien-6-one | 0.297 | 0.465 | 0.001 | 0.710 | 70.964 |
| 350 | (10E,15Z)-9,12,13-Trihydroxy-10,15-octadecadienoic.acid | 0.189 | 1.372 | 0.000 | 0.981 | 98.148 |
| 351 | Abscisic acid | 0.222 | 1.674 | 0.000 | 0.983 | 98.277 |
| 352 | Discodermolide | 0.058 | 1.582 | 0.000 | 0.999 | 99.865 |
| 353 | Galanthamine | 0.126 | 1.320 | 0.000 | 0.991 | 99.094 |

**Supplementary Table 2**. Raw intensities of the 353 metabolites identified as differentially abundant between the 4 collection time points [T1, T2, T4: 1, 2 and 4 minutes, respectively, after tying the tourniquet and NT (no tourniquet with collection 5 minutes after removal of the tourniquet)] in 05 study participants. Samples have been coded as: Subject ID Timepoint [for example, S2T1 represents data for subject S2 and timepoint T1].

**Supplementary Table 3**. Pareto-scaled intensities of the 353 metabolites identified as differentially abundant between the 4 collection time points [T1, T2, T4: 1, 2 and 4 minutes, respectively, after tying the tourniquet and NT (no tourniquet with collection 5 minutes after removal of the tourniquet)] in 05 study participants. Samples have been coded as: Subject ID Timepoint [for example, S2T1 represents data for subject S2 and timepoint T1].
